# Political entrepreneurs in social media: Self-monitoring, authenticity and connective democracy. The case of Íñigo Errejón

**DOI:** 10.1016/j.heliyon.2023.e13262

**Published:** 2023-01-27

**Authors:** Maria Iranzo-Cabrera, Andreu Casero-Ripollés

**Affiliations:** a Department of Language Theory and Communication Sciences, University of Valencia, Valencia, Spain; b Department of Communication Sciences, Universitat Jaume I, Castellón, Spain

**Keywords:** Political communication, Social media, Digital, Accountability, Twitch, Monitory democracy, Deliberative democracy, Connective democracy

## Abstract

Political entrepreneurs seek to mobilise public opinion and access large audiences who are not directly interested in politics, but are exposed to the digital environment. The aim of this research was to analyse how these figures promote experimental communication uses on channels far removed from political activity. We focused on Twitch, a successful platform for promoting entertainment and learning in the video games field. To do so, we conducted a significant case study, that of Íñigo Errejón, a Spanish male Member of Parliament, in 2021 through 18 live streamings that lasted 1223 min. We specifically described the conception and use of Twitch, measured the audience's impact, analysed the accountability exercise through this platform and evaluated the deliberative quality of conversation with users. To conclude, we identified three novel contributions of Twitch to digital political communication: self-monitoring, insofar as the elected politician himself proactively exercises accountability to the public without a third party intervening; the activation of mediated authenticity as a key value in the political actor's public construction; promoting connective democracy, which would help those sectors not used to employing political information to take an interest in it by detecting attention being paid to their needs and questions.

## 1 Introduction

On January 29, 2021, Íñigo Errejón, a Spanish male Member of Parliament, a founder of the Podemos political party, and now a member of the Más País political party, started broadcasting via his Twitch channel (https://www.twitch.tv/ierrejon). He was the first Spanish politician to do so and was, internationally, one of the first Members of Parliament to use this platform. Earlier in October 2020, US congresswoman Alexandria Ocasio-Cortez had employed this digital means, but only sporadically. Since October 2018, French politician Jean-Luc Mélenchon has had a Twitch account where he has occasionally broadcast public events, made unidirectional messages and answered citizens via chat, but not frequently. When Errejón started using this platform, he stated: “Official politics always tends to be separated from the real world because, in the end, politicians and journalists only talk to one another. Using the phone to be able to interact with people to expose your fears or criticisms is a reality check that I believe is enriching” [1]. Along with this important declaration of principles, Errejón's decision involves a highly innovative component: employing this live streaming service, which is preferentially oriented to video games, and to promote other ways of doing politics.

This reveals how politicians are utilising digital means to reach the public with their positions and policies. These platforms allow new strategies, new practices, new actors and new contents to emerge, which are changing how political information is produced, distributed and used [2]. These processes influence politics because they generate many effects that influence how democracy works and open up a field for experimentation. The depth and importance of such changes mean that social media are retooling politics in contemporary democracies [3].

Digital platforms have three essential characteristics for this purpose: interactivity, connectivity and accessibility [4]. First of all, they offer the chance to communicate to one another by driving exchanges and dialogic practices. By means of the many-to-many communication model, these platforms allow the capacity to respond, or feedback, which offers the possibility of generating interactivity. Nevertheless, former research works have demonstrated that politicians scarcely use and interact with digital technologies [5]. Likewise, social media favour connectivity among citizens which, thanks to these platforms, can establish links with other people, and allow them to collaborate or connect with one another.

Accessibility refers to the capacity to employ contents, but to also produce and distribute them. Digital technologies have drastically cut the costs of creating, producing and distributing them. Any user can create and disseminate his/her own contents autonomously without intermediaries. This favours a more open and decentralised scenario where competition to control the information flow is fierce [6]. The marked leading role of news media and journalists as gatekeepers in relation to the information that reaches the public is weakening because numerous voices in the digital realm have emerged. Political actors have started opening accounts with Twitter, Facebook, YouTube, Instagram and other platforms to directly reach large audiences. Apart from conventional political parties, this has allowed a series of emerging actors to look at social media and to actively perform in these spaces. They are political entrepreneurs [7] who seek to mobilise public opinion and to access large audiences that are not directly interested in politics, but are exposed to the digital environment. To this end, they make a basic element of their activity out of their highly innovative communication strategies on digital media [8].

The literature has focused on demonstrating that politics use social media to diffuse information, but has paid less attention to political entrepreneurs' innovative communication practices and their democratic consequences [9]. To bridge this gap, we focus on how these politicians drive the experimental communication uses of digital platforms that are far-removed from political activity, such as Twitch. For this purpose, we analysed Íñigo Errejón's channel on this platform throughout 2021 by means of 18 live streamings, which collectively lasted 1223 min. There are three reasons for choosing this case study. The first one is because he is the only politician with a seat in Parliament to use this tool in Spain, and one of the few Members of Parliament who do so internationally. So, we can consider him a political entrepreneur. The second reason is that he frequently uses this platform. Other politicians like Alexandria Ocasio-Cortez have also employed this digital means, but not so intensely. The third and final reason is his pioneering and innovative experience because he employs a platform that is more oriented to video games and e-sports for a political use. These three reasons make this case study interesting and relevant for knowing political actors' communication practices on Twitch and their democratic incidence. This research work specifically takes the case study of Errejón to: detect and describe the conception and use of the Twitch channel; measure the impact on the audience; analyse the accountability exercise by means of this platform; evaluate the channel's deliberative activity.

## 2 Literature review

### 2.1 Social media, connective democracy and political entrepreneurs

Social media possess specific affordances that allow new communication dynamics to be activated [4], that are capable of potentially transforming politics and exercising power [10]. For example, users being able to autonomously generate and distribute contents to third parties, and having the capacity to establish connections among citizens or to generate cooperation mechanisms with legacy media by shaping a hybrid and high-density communication environment are some of the main affordances [6]. Several previous research works have analysed the impact that these digital platforms have on ways of doing politics by detecting different effects [2]. Some works have demonstrated the potential of social media for citizen activism and for encouraging political participation [[11], [12], [13]] to introduce new ways to build the agenda [14] or to make new communication strategies emerge [8]. Other research works have, conversely, revealed that these digital media have favoured the new leading role of old problems, such as disinformation [15,16] or polarisation [17,18], by contributing to produce disruptive public spheres [19] that are becoming increasingly fractured [20].

In this context, our democracy is witnessing a redefinition process. This is also being driven by a growing disconnection between citizens and democratic institutions that reinforces mistrust and disappointment of formal politics [21]. Political actors are perceived as a privileged class who have no idea about common people's problems and concerns. This public discontent scenario has encouraged extremism and populism to grow in different parts of the world [22].

Social media are ideal tools for generating new connections among citizens. Given their affordances, these platforms are extremely capable of creating links among individuals and of cementing relationships with them [4]. To this end, they are fundamental for promoting connections in the public sphere. Such connections are a basic ingredient for democracy, and act as the basis for forming a political community [23]. Indeed previous research works have identified several formulae by means of which digital platforms are capable of generating political connections with a democratic value.

The first one is the monitoring involved in activating processes to oversee political, economic and media power [24]. Abundant information favoured by an online setting and advancing with transparency in the political field mean that any citizen can access a large quantity of data, which can be used to scrutinise social elites' actions and decisions. Moreover, social media are converted into a channel by means of which denunciations of abuses of power can be quickly and easily diffused. The fact that digital platforms are open in nature means that this monitoring action can also be done by citizens for a civic purpose [25]. This dynamics, which is related to the monitory democracy concept [26], results from lack of trust in and disliking institutional power. Its activation places the focal point on accountability, which aims to ensure power's responsible performance, which is capable of exposing, justifying and publicly defending their decisions. This practice has the potential to connect citizens to democracy [27] because it favours the public's empowerment. In the last decade, social media have led some examples of monitoring in several countries to emerge, including Spain [28].

The second one is political actors' humanisation. Digital platforms allow these leaders to promote the self-mediation of their communication by directly addressing citizens without intermediaries. This enables the possibility of activating different communication strategies to connect to the public. One of them consists in showing certain political leaders' intimate aspects, such as personal details of their lives, their likings or hobbies, their everyday habits or their emotional facet [29]. In this way, they create illusions of authenticity that make them appear to be more realistic, genuine and trustworthy because they employ the characteristic elements of mediated authenticity, such as spontaneity, immediacy, imperfection, confessions or ordinariness [30]. This not only allows a direct relationship based on closeness with the public to be established, but also cuts the distance between citizens and elected representatives. This especially occurs when politicians “have insight into voters' demands and feelings at a time such as the present when the declining influence of party politics and traditional ideologies has left avoid in voters' allegiances” [31]**.** So, it could foster attachment and trust [32] by improving the public's connection with democracy. This strategy is quite normal with populist politicians given their intention to represent “the people” and defend common people's interests [33].

The third one involves promoting the discursive spaces that favour constructive deliberative debate. Despite its interactive potential, as previously mentioned, social media have contributed to drive social and political divisions by stressing affective polarisation [17]. Moreover, political actors have mostly denied using these digital platforms to encourage dialogue with citizens. Conversely, a conception based on one-way communication has predominated, which shapes these media as channels that distribute information and contents, but barely interact with the public [2]. This has enhanced the disconnection between politics and citizens in the digital setting. Furthermore, previous research into citizens’ comments posted on social media evidences very little discussion of a rational kind [34]. Two determining factors have an influence here: homophily and selective exposure [23]. Citizens tend to avoid cognitive dissonance [35], and usually follow those with whom they share an ideological, cultural or behavioural link. This creates informative bubbles or echo chambers that generate polarisation [36,37], although some authors have questioned its effects [38].

To revert this situation, some authors propose promoting connective democracy [39]. Here the objective is to generate quality connections that allow opinions and pieces of information to freely flow, bidirectional public discussion and respect for the other person as a member of the same political community [23]. The intention here is to solve polarisation and disconnection problems by listening and deliberation [40]. In this way, connective democracy is linked with the Habermasian public sphere notion [41], which understands political deliberation with interactions sustained in discursive diversity, argumentation and reciprocity that derive from the social action of listening. Meeting these three standards means that political conversations move towards a deliberative ideal [42] and, by doing so, towards a consensus being reached. The principle of parrhesia is also a political actors’ obligation; that is, speaking the truth [43].

These three monitoring, humanisation and connective democracy formulae are laid down on daily, intentional and creative practices that seek to renew political connections among citizens by developing a mending democracy dynamics [44]. When activating them, political entrepreneurs play a crucial role because they are capable of not only mobilising resources and emotions to impact citizens, but of also activating the public's reactions. These actors work on a small scale to re-establish citizens' links with different parts of the democratic process by applying ingenious forms of engagement [45]. To accomplish this, they apply innovative communication strategies, such as resorting to new digital formats of contents to diffuse their messages [7], and building an agenda that focuses on new and alternative themes [46], which are excluded from news organisations or the criticism of the establishment. They are vectors that bring about political change to make democracy strong or weak. Despite their importance, studying these political entrepreneurs is a poorly explored area. Therefore, we propose studying a significant case to advance in knowledge about not only the role of these figures in the digital setting, but also about their capacity to create connections and to mend democracy.

### 2.2 Twitch: a digital live streaming platform

Twitch is a digital live streaming service that first appeared in 2011. Amazon has owned it since 2014 when it purchased it for 970 million dollars. Its content preferentially focuses on video games and e-sports, although it is potentially open to all kinds of themes. In fact according to TwitchTracker, its “just chatting” content has increased by 200% since 2021 with 15% of viewers. In all, it has 7.1 million active channels, its average number of daily visits is 31 million and it used up 1.3 trillion minutes in 2021 (https://www.twitch.tv/p/press-center/). Its public profile is mostly young people because 75% of users are aged from 16 to 34 years, who form part of Generation Z.

Twitch had increased its social impact in recent times. During COVID-19, its number of users grew by 200%. This, along with the growing interest in video games and e-sports, plus the consolidation of outstanding streamers (https://socialblade.com/twitch/top/50), or content creators like Ninja (with 18.8 million followers), Auronplay (14 million), Rubius (12.8 million) or Ibai Llanos (11.7 million), have encouraged Twitch usage worldwide.

In accordance with Spilker and Colbjørnsen's [47] characterization of streaming services, Twitch offers user-generated, live, on focused and general-audience streaming. This platform is based on three traits: immediacy, interaction and generating a community around a given passion, which could be related to video games, a sport, cryptocurrencies or politics. First of all, and unlike YouTube, contents can only be created live and edited products cannot be diffused. Anybody with certain technical knowledge and, above all, anyone with a rapid Internet connection, can broadcast. However, Twitch requires not only ensuring quality live streaming, but also the skill to broadcast authenticity [30]. A streamer, or a content creator on Twitch, must know how to express oneself, talk and communicate “in a manner relevant to the subculture one wishes to engage with” and must do so “easily and comfortably” [48]. Talking enthusiastically about what one knows and likes is an outstanding quality on this digital platform.

The contents on Twitch appear live and are perishable (they disappear a few days after being broadcast), which encourages users to wish to personally witness streamings. Twitch channels fit the “third places” description [[49], [50], [51]]. Such spaces periodically host meetings. They are voluntary and informal, and are happily anticipated by individuals beyond their homes or workplaces. Nonetheless, hybrid logics operates between Twitch and YouTube [52] insofar as the latter acts as a repository of streamers to multiply diffusion [53]. This avoids contents disappearing, and also extends their useful life and their diffusion.

The second requirement for content creators, and Twitch's true challenge, is to know how to manage both their intervention and the audience's attention and satisfaction in real time [54]. For this reason, as they gain followers, they make up a team of moderators (with at least three or four people) that can be in charge of performing and controlling the chat to particularly avoid trolls intervening because they block conversation. On this matter, different research works point out the positive effect of playing this role in achieving deliberative quality, provided that it acts responsibly [55,56].

The user's interface and experience are designed for the audience to participate live. For this purpose, the chat occupies a relevant place on the screen. Indeed to favour interaction, it is possible to slow down the comments that appear so that the streamer “can realistically expect to read every message they get sent, reply to them, and build a sense of community” [48]. For Hilvert-Bruce et al. [57], content creators who broadcast over small channels (with fewer than 500 followers) come over as being more motivated to make social engagement by addressing themselves using participants' names. Therefore, Twitch integrates a double screen [58]: conventional live streamings (broadcast media) and interaction by means of chat (social media). True engagement appears on the latter screen [59].

To date, research into this platform has centred mostly on the uses and gratifications that it generates for the so-called gamers [48,60]. Essentially, entertainment and learning [61] have been identified as the main claims for live viewing, as well as the building of an online social community via chat: “viewers express support, opinions, suggestions, requests, even criticism via chat in real-time as the streamers produce content tailored to their live audience” [62]**.** Streamers' motivations are the communicative interaction and the audience's commitment [63]. Ask et al. [64] propose five dimensions that determine the user-platform relationship in Twitch. These are: 1) Sociality: community or individual use; 2) Audience: specific or general; 3) Moderation: strictly moderated or laissez-faire; 4) Content: user-generated or commercial; 5) Scope: specialized or multi-feature.

As an exception we should point out the exploratory study that Ruiz-Bravo et al. [65] have made of the usage of live chats in e-games channels for promote political activism, related to the protests in Hong Kong in 2019. However, Twitch remains unexplored from the point of view of political communication and its connection with democracy.

Its potential as a political communication tool has been proven by US congresswoman Alexandria Ocasio-Cortez, who is popularly known by her initials as AOC. She opened her account on March 23, 2020, but her first streaming did not take place until 20 October. This democratic lady politician has had three live streamings. The first two were video game sessions, which drew 430,000 simultaneous spectators. The third was used to explain and contextualise the company game STOP's rise on the stock exchange. Several experts intervened in this live streaming, who simply explained the world of the stock exchange and shares. Ocasio-Cortez did not attempt to replicate the strategy and type of messages from other social networks on Twitch, but gave a differential value to the way to communicate on this platform. Nevertheless, her experience was limited and sporadic. The naturalness with which she dealt with the mistakes made while broadcasting and extending live streamings “contrasts with the figure that is usually associated with an immaculate politician who must never make a mistake” [66].

Jean-Luc Mélenchon, the leader of the La France Insoumise (France Unbowed) political party, has used this platform to broadcast the party's events since October 4, 2018. Gifted with “his talent as a speaker”, according to Bonet [67] one of the reasons for his success in the April 2022 presidential elections “was young people's support, and for being the most voted person by voters aged from 18 to 34 years”. Influencers asked for the eco-socialist leader's vote, who ended the campaign with a long streaming via Twitch. Earlier he had also used this platform to answer the audience's questions, which he did on six occasions: May 29, 2020 (2 h, 11′), July 16, 2020 (2 h, 15′), November 8, 2021 (1 h, 53′), February 1, 2022 (1 h, 24′), April 8, 2022 (1 h, 24′) and June 17, 2022 (1 h, 21′). His website includes a repository of the last three streamings (https://melenchon.fr/categorie/videos/allo-melenchon/), which were broadcast from a set with two moderators. He identifies this format with the hashatg #AlloMelenchon.

Nonetheless, the political use of Twitch for accountability has been limited to only a few sporadic cases. One of the most interesting ones is that led by Íñigo Errejón, a Spanish male Member of Parliament. This political entrepreneur has frequently used this digital platform throughout 2021. We pose the following research questions.

RQ1: How does Íñigo Errejón use his Twitch channel (streaming frequency and themes) and how does it impact the audience (viewings, followers and spectators)?
RQ2: How is the political actor's construction formed on Íñigo Errejón's Twitch account?
RQ3: How is accountability implemented on Íñigo Errejón's Twitch account?
RQ4: What deliberative characteristics do the creator-audience dialogic debate and the forms of engagement that are employed in Íñigo Errejón's Twitch account have?

## 3 Materials and methods: four analysis dimensions

**Fig. 1:**
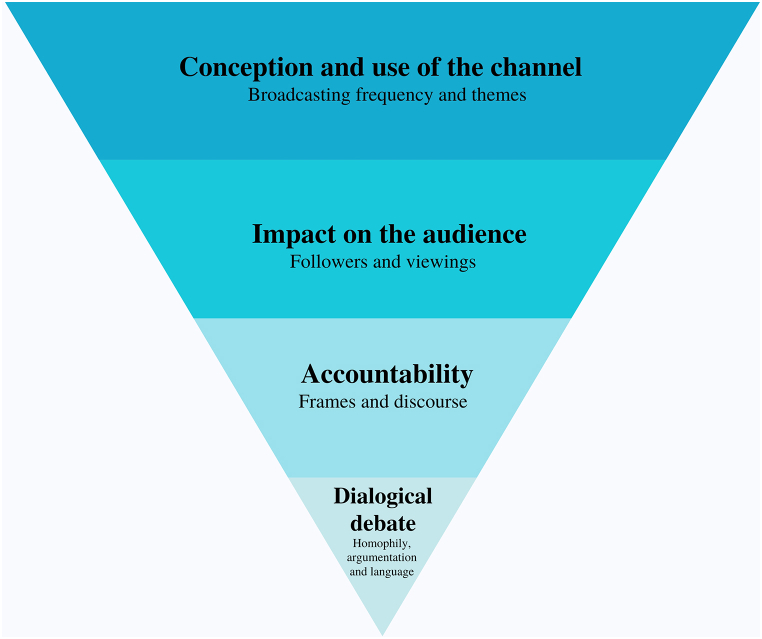
The analytical dimensions of this research. Source: the authors.

The present research work applies the case study methodology, which implies analysing “exhaustively a singular case interpreted in particular socio-political contexts” [68]. Íñigo Errejón is the only leader of a political party with parliamentary representation in Spain who has used this streaming platform to continuously communicate with his followers over time. This makes him a political entrepreneur because he has included innovations in his political communication formulae to relate to citizens. As this is a “case in action” [69], an approach strategy was designed with four analytical dimensions (Fig. 1) that answer the research questions from more general aspects to more specific ones, and also from quantitative data to qualitative ones. Each analytical dimension respectively requires an interpretation method and a specific sample, and they are all related to the studied case.

The conception and use of the channel is dealt with on the first analytical dimension. To do so, the descriptive observation of dates, times and duration of streamings, and of the 18 live streamings themes by Íñigo Errejón channel in 2021, was carried out (Table 1).

**Table 1:** Dates, times and durations, plus streaming theme.

Date	Month	Day of the week	Title	Streaming type	Duration (in minutes)	Start time	End time
30	November	Tuesday	Meritocracy	alone	40	18:37	19:17
18	October	Monday	Talk with Inés Hernand	dialogue	85	16:03	17:28
30	September	Thursday	We talk about the Law on Cannabis	alone	31	12:33	13:05
7	September	Tuesday	We return to Twitch	alone	44	12:21	13:05
4	May	Tuesday	Talking live #MásMadrid4M	discussion	31	17:24	17:55
2	May	Sunday	Campaign closure!	discussion	27	19:08	19:35
1	May	Saturday	We're in Móstoles!	discussion	86	18:49	19:15
29	April	Thursday	Video games, e-sports, economy and culture with Ángel Quintana	dialogue	66	13:09	14:15
23	April	Friday	Talking with Javier Giner about his book ‘Me, an addict'	dialogue	73	13:22	14:35
21	April	Wednesday	Daily LGTBIphobia with Víctor Gutiérrez	dialogue	70	19:15	20:25
16	April	Friday	Campaign ideas	discussion	74	13:11	14:25
6	April	Tuesday	Talking with Mónica García about mental health	dialogue	72	13:23	14:35
25	March	Thursday	Talking live from Parliament	alone	52	17:13	18:05
12	March	Friday	With Elizabeth Duval	dialogue	114	13:01	14:55
25	February	Thursday	Talking about mental health from Parliament	alone	63	13:12	14:15
19	February	Friday	No title	alone	90	20:14	21:45
4	February	Thursday	No title	alone	117	20:38	22:35
29	January	Friday	No title	alone	88	19:57	21:25

The second analytical dimension explores the impact on the audience by means of the quantitative data recorded by the TwitchTracker and SullyGnome monitoring applications in the sample found in Table 2. The following variables specifically apply: mean number of spectators, peak of spectators, gained followers and viewings.

**Table 2:** Impact on the audience according to TwitchTracker (T) and SullyGnome (S).

Title	Spectators (mean) T	Spectators (mean) S	Spectators (peak) T	Spectators (peak) S	Followers gained T	Followers gained S	Viewings T	Viewings S
Meritocracy	286	286	338	338	53	53	280	280
Talk with Inés Hernand	564	566	609	609	63	57	571	571
We talk about the Law on Cannabis	290	311	348	348	34	22	254	254
We return to Twitch	269	249	323	336	40	40	391	391
Let's talk live #MásMadrid4M	1279	1161	1664	1846	334	269	1955	1955
Campaign closure!	442	396	566	566	31	48	0	0
We're in Móstoles!	412	362	462	362	33	0	0	0
Video games, e-sports, economy and culture with Ángel Quintana	794	592	971	971	363	339	1575	1575
Talking with Javier Giner about his book ‘Me, an addict'	411	405	464	482	204	215	1542	1542
Daily LGTBIphobia with Víctor Gutiérrez	379	403	153	453	153	119	853	698
Campaign ideas	311	264	337	349	56	60	442	442
Talking with Mónica García about mental health	485	487	647	647	123	124	355	843
Talking live from Parliament	497	553	646	714	233	204	274	0
with Elizabeth Duval	515	530	636	662	194	188	1579	1407
Talking about mental health from Parliament	817	672	1037	1037	442	393	1968	1968
No title	492	519	673	581	188	172	725	725
No title	524	549	634	634	377	396	1986	1986
No title	875	900	1032	1032	641	556	968	968

The third analytical dimension focuses on accountability. The employed method is the analysis of the qualitative discourse of the contents on Errejón's Twitch channel. The sample is made up of 10 live streamings (Table 3). The long duration of these live streaming (each one lasting about 60 min) is the reason why we decided to work on this dimension with 55.55% of the population. For the analysis, a transcription was done of 552 min. With the Atlas.ti programme, the frames by means of which the political actor defines the contents broadcast on this digital platform were observed. For coding, the classification of the four main theme categories taken from Patterson's typology (1980) was used. They encompass the 109 detected frames. The four main theme categories were.

- *political issues*: questions relating to the party's ideology, alliances with parties, and relations with civil society and the groups governing at the time
- *sectorial policy issues*: health, education, economy, etc.
- *personal issues*: issues related to candidates' life and activities, their character and hobbies, among others
- *campaign issues*: matters related to the organisation of electoral campaigns; in our case, the Madrid campaign (campaign events, surveys, candidacies)

**Table 3:** Accountability sample.

Date	Month	Title	Participants
30	November	Meritocracy	Íñigo Errejón
18	October	Talk with Inés Hernand	Íñigo Errejón and Inés Hernández (Inés Hernand)
30	September	We talk about the Law on Cannabis	Íñigo Errejón
7	September	We return to Twitch	Íñigo Errejón
23	April	Talking with Javier Giner about his book ‘Me, an addict'	Íñigo Errejón and Javier Giner
21	April	Daily LGTBIphobia with Víctor Gutiérrez	Íñigo Errejón and Víctor Gutiérrez
16	April	Campaign ideas	Íñigo Errejón, Gala San Migule, Gabi Ortega, Daniel Ayuso, Ana González and Clara Menchén
6	April	Talking with Mónica García about mental health	Íñigo Errejón and Mónica García
25	February	Talking about mental health from Parliament	Íñigo Errejón
29	January	No title	Íñigo Errejón

Finally, the fourth analytical dimension studies the creator-audience deliberative debate and the employed engagement forms. To do so, a qualitative discourse analysis was carried out of the audience during the chat of two live streaming that were broadcast at the beginning and end of 2021 (Table 4). They were chosen because one was the live streaming that aroused more interest and centred on Errejón's most applauded speech from the Parliament Stand, and the other was that which the leader gave on a controversial matter: appointing Marta Ortega as Chair of Inditex and meritocracy in Spain. Both speeches were analysed by manual coding and by applying the variables below [34] to analyse the democratic value of both these political conversations.

• Theme. This variable evaluates coherence in conversation. The participants should reflect mainly on the themes that this political leader also addresses during his speech
• Homophy. It is defined as an ideological affinity between the political actor and the participants. Comments were coded by these categories: a) against, b) neutral and c) in favour of the opinion that Íñigo Errejón expressed.
• Rationality of arguments. This was measured by detecting those comments that included theoretical citations, and also those with an argument (those that used causal conjunctions or explicitly included data or evidence to sustain his statement). All these comments were based on fallible knowledge
• The reciprocal interaction. During conversation, the participants show certain willingness to listen to the ideas and views of the other fellow participants, and to exchange questions and reasons. This category is operationalised by means of five indicators: a) number of interventions in the chat; b) direct answers or mentions made by the leader to comments; c) number of repeated comments (evidence for the speaker's disregard and him insisting on seeking an answer); d) number of questions asked; e) use of vocatives (reflecting the desire to interact)
• Respectful discourse. This analyses the type of language that commentators use according to these categories: a) respectful, neutral language; b) offensive language (insults, capitals); c) offensive language addressing certain people. Given the widespread use of iconography and some comments being made only with emojis, a distinction was also made between positive emojis (they convey supportive messages and are, therefore, taken as respectful, neutral language) and negative emojis (they convey aggressive messages and are, therefore, taken as offensive language)

**Table 4:** Sample used to analyse the democratic value of political conversations.

Date	Month	Title	Spectators (mean)	Spectators (peak)	Participants in the chat	Messages sent
30	November	Meritocracy	286	338	147	421
25	March	Talking live from Parliament	525	680	451	1122

Apart from these five variables, quantitative data were obtained: user name and; if it was of the Twitch ‘premium’ condition (paying the platform 3.99 euros a month to enjoy certain advantages, such as more viewing during his speeches), in order to confirm if this meant more presence during dialogue; followers' age, their profession and location if explicitly made known in comments.

The process of collecting data and coding Íñigo Errejón's speeches was followed by the same researcher descriptively to reduce the risk of subjective interpretation in this analysis stage. In the second phase, two researchers worked together to interpret the collected data.

## 4 Results

### 4.1 Conception and use of the channel

**Fig. 2:**
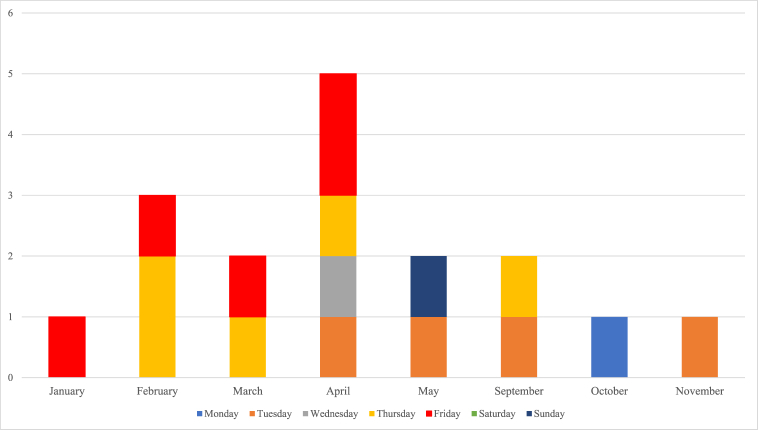
Íñigo Errejón's live streamings on Twitch in 2021. Source: TwitchTracker.

Íñigo Errejón started broadcasting on Twitch on January 29, 2021, and his last streaming started on November 30, 2021 (Fig. 2). During this period, he did 18 live streamings, which can be classified as these three types: interactions alone and with an audience (44.5%, n = 8); dialogue with guests (writer Elizabeth Duval, Más Madrid party candidate Mónica García, water polo player Víctor Gutiérrez, producer Javier Giner, professor of History and Cinema Theory Ángel Quintana and communicator Inés Hernand) (33.3%, n = 6); talks held with Más Madrid Party candidates and members as part of the local Madrid elections (22.2%, n = 4).

In accordance with the five dimensions that determine the user-platform relationship proposed by Ask et al. [64], Errejón's Twitch channel should be defined by its community use, general audience, moderated chat, user-generated and specialized purpose: reconnecting the politician with the interests and concerns of the citizenry.

Despite the fact that during his first streamings Errejón mentioned that he would do one streaming “at least every fortnight”, this actually occurred only between February and April. After the Más Madrid electoral campaign in the Madrid Community (May 4, 2021), he did none for 4 months. He organised four more live streamings between September and November.

The days when plenary sessions are held in Parliament (Tuesdays and Thursdays), and also Fridays when Parliament has no organised events, were the days when Errejón did more live streamings (totalling 77.7% in all). Of the total, 55.5% (n = 10) were scheduled in the late afternoon/evening (15:00–22:00 h) and 44.5% (n = 8) in the morning/early afternoon (8:00h-15:00 h). The latter streamings were scheduled at around 13:00 h. Their average duration was 68 min each.

Regarding themes, 33.3% (n = 6) had no specific theme, 27.8% (n = 5) were about mental health (including the management of LGTBIphobia or addictions) and 22.2% (n = 4) were used as a political programme for his party Más Madrid. Apart from these, there were three other streamings (16.7%) about sectorial topics: one to talk about his proposal to legalise cannabis, another to question meritocracy in Spain and a third about the video games and e-sports sector.

### 4.2 Impact on the audience

**Fig. 3:**
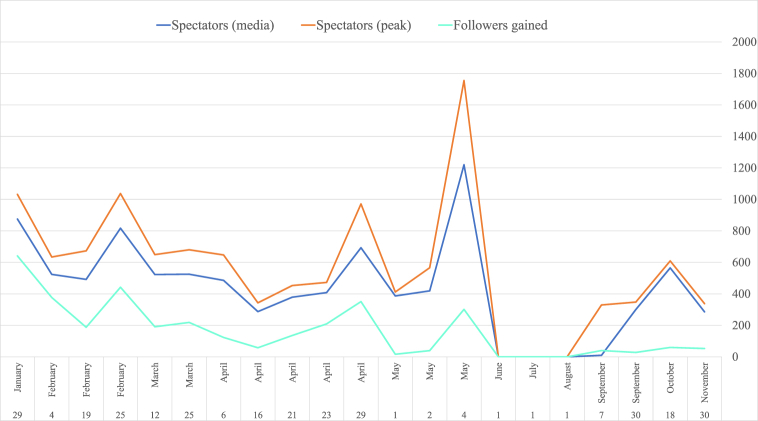
Spectators for the @ierrejon streaming on Twitch in 2021. Source: TwitchTracker and SullyGnome.

Errejón's Twitch channel attracted a mean of 523.53 simultaneous spectators in 2021, according to the average of the data recorded by TwitchTracker and SullyGnome. A mean of more than 700 followers listened to three speeches: his first live streaming (21 January, 887.5), his broadcast on the day after the speech he made from the Parliament Stand about paying more public attention to mental health (25 February, 744.5) and on the day that the local Madrid Community elections took place (4 May, 1220). His audience peaked during these three live streamings. The maximum peak was 1755 followers (4 May). Not meeting a set streamings frequency influenced the number of followers. During the last 4-monthly period, when his speeches were more spread out in time and after no streaming in summer months, the peak did not reach his mean number of followers at the beginning of the year: 352.5 followers *versus* the mean of 572.35 followers recorded from January to May (Fig. 3). Therefore, the data indicate more public attention at the beginning than at the end of the studied period.

The increase in the number of followers of this channel was greater for the first four live streamings (more than 350 new followers per streaming). Their number did not change on May 4, 2021 with 11,938. Therefore in 4 months, his followers increased by 425.44%. In December 2021, the community came to 13,655. The resulting mean was 189.36 new followers per live streaming.

There was a mean of 870 single viewings for each live streaming. Three live streamings stood out with around 2000 viewings: his second live streaming about several themes (1,986), the one about defending mental health in Parliament (1,968) and his live talk #MásMadrid4M (1,955). Conversely with no viewings, there were two live streamings that focused on the electoral campaign. This demonstrates that the broadcasts dealing more with electoral persuasion attracted less citizen interest.

### 4.3 Accountability

During his first live streaming on January 21, 2021, Íñigo Errejón justified using this channel as a “public server's” “bidirectional communication” means with society; “a kind of contact without filters or intermediaries”. For this politician, “making this effort to go out and listen” is necessary to know the “problems, questions, concerns, desires or themes on the street that perhaps do not reach institutions”. The following are pointed out as the advantages over social networks:

@ierrejon: “This [accountability] should be practiced by more Members of Parliament because it helps to explain what is done, to justify reasons, to accept critical questions” (29 September 2021).

@ierrejon: “Live streaming like this gives you some opportunities that the rest don’t, with things you don’t have to explain in just two sentences because here you can expand on things a bit more” (30 November 2021).

In the conception of this digital platform as part of Errejón's communication strategies, orientation towards accountability is detected. Errejón conceives his Twitch channel as a way to practice monitory democracy [25,26] because he seeks to supervise public representatives' actions as, in his case, a Member of Parliament. As this was driven by him, we could call it self-monitoring. With this digital means, he intends to provide explanations about his proposals and political decisions by applying the principle of transparency by submitting himself to citizens' scrutiny.

In order to analyse how accountability is put into practice on this Twitch channel and to answer RQ3, the frameworks of Errejón's speech were taken by analysing 667 citations (Table 5) that fall in four categories (campaign issues, sectorial polity issues, personal issues and political issues). The data indicate considerable relevance attached to sectorial policy issues (49.33%). In fact, Errejón diffused his position about social (36.39%), economic (32.92%) and health (30.69%) issues over this digital channel, which he himself defines as “the problems that matter to people”. To provide them with a solution, he framed different the Más País party initiatives that were presented in Parliament within those related to mental health and cannabis.

**Table 5:** Frameworks of Íñigo Errejón's speech on Twitch. Source: Atlas.ti

	**21012021**_First live streaming on Twitch	**20092021**_ We talk about the Law on Cannabis	**30112021**_Meritocracy	**19102021**_Errejón and Ines Hernand	**25022021**_ Talking about mental health from Parliament	**23042021**_ Talking with Javier Giner about his book ‘Me, an addict’	**21042021**_Daily LGTBIphobia with Victor Gutierrez 2	**06042021**_ Talking with Mónica García about mental health	**07092021**_We return to Twitch 2 de 2	**16042021**_Campaign ideas 2	Total	Relative total
Campaign issues	0	0	0	0	0	1	0	4	0	21	26	3.90%
Sectorial policy issues	43	43	43	30	80	22	26	20	6	16	329	49.33%
Personal issues	75	7	4	4	21	7	3	2	1	6	130	19.49%
Political issues	37	24	19	3	44	10	16	9	8	12	182	27.29%
											667	100%

Specifically 102 ‘mental health’ citations were found in his discourse, which represent 15.3% of the total. Throughout practically all the analysed speeches, Errejón underlined that his political party has been the only one to have “opened a gap” in a problem that had been silenced until the present-day and one that had particularly flourished during the COVID-19 pandemic. This reveals that this politician employs Twitch to attempt to promote this theme on the political and social agenda. Regarding frequency of use, these codes follow this: ‘cannabis’ and ‘Más País’ (5.5%, n = 37), ‘the common good’ (5.1%, n = 34), ‘public health’ (4%, n = 27), ‘public services’ (3.6%, n = 24) and ‘social networks’ (3.6%, n = 24).

The personal issues category represents 19.49% of the total. On his likings and based on the questions he was asked, he particularly referred to his musical (2.1%, n = 14) and gastronomic (1.9%, n = 13) preferences. Addressing such of issues in the contents broadcast by Twitch is connected with the aim to humanise this politician's public figure. On political issues, he stated that he does not smoke cannabis, he does not visit a psychologist and he found it easy to learn Catalan, which is the language he uses to answer any questions made in this language. When asked if he made money with the platform, which he cannot because his account is not configured to receive donations, Errejón emphasised once again his self-monitoring orientation:

@ierrejon: “This forms part of my work as a Member of Parliament. Of course, I don’t make money with Twitch, I do so by accountability and explaining myself. I don’t know who you vote for, but I hope you vote those who also do this. Let’s hope that the Member of Parliaments you vote for answer questions like these and discuss such things” (56′25 | 25 February 2021)

**Fig. 4:**
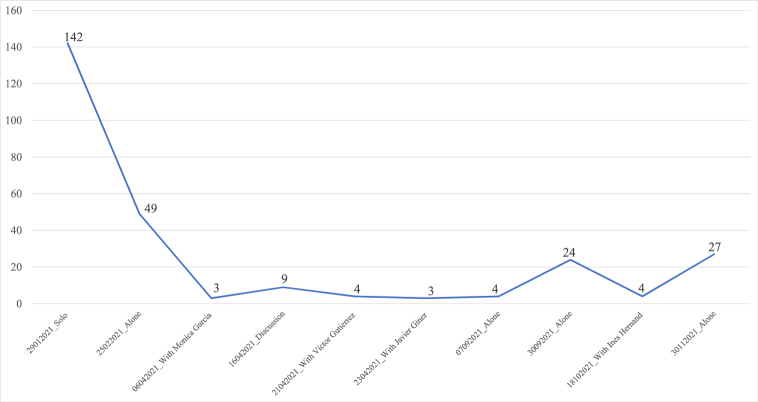
Absolute number of answers to comments given by Íñigo Errejón during each live streaming on Twitch in 2021. Source: the authors.

The intensity of his first live streaming, during which 142 answers to comments made during the chat were recorded, became more diluted over the study period. The live format impacts the fact that his interaction is more or less intense; that is, whether Errejón participates only in streamings or opts for the talk/interview or guest/discussion formats. During the live streamings he did alone (Fig. 4), 91.44% (n = 246) of the all the answers he gave to comments (n = 269) were concentrated. However, during the interview and talk formats, the leading figures were the guests involved and Errejón acted as a moderator without making his accountability commitment (n = 23).

### 4.4 Deliberation and dialogic debate

To answer RQ4, we detected that the publication rate in the chat of the analysed broadcasts was a mean of 14.35 comments/minute (18.7 during his first live streaming and 10 during his second). In them 598 single accounts participated (451 and 147, respectively) and more than half the users had no “premium” account (64.96% and 63.26, respectively).

During both the above-cited live streamings, comments were about the political leader's main discourse themes: mental health (39.35%) and meritocracy (51.56%). During both, the Twitch-related technical issues (production, moderators) were the second most important theme, but presence was considerably lower (10.75% and 6.51%, respectively).

Sometimes users thanked him for using this platform to encourage conversations with citizens and asked him to use it periodically and regularly:

@fuitetcey: “I think that this format is an excellent exercise to come close to citizens. Let’s see if you can convince other Members of Parliament to do the same and we make you all get your act together a bit more rather than the mass media talking about what they want”.

@royit00: “Talking about mental health on a streaming platform, adapting to the mass media and to people’s needs and problems, also form part of politics”.

FlapsyGames: “Are you thinking about doing these streaming in the long term? During the pandemic you said it was compulsory and you’d do one every week. I'm not saying you’re very busy, but doing this regularly would be a good thing”.

On homophily, the messages in favour of Errejón's speech were 55.71% during the streaming about mental health and 42.37% during that on meritocracy. Neutral speeches were 34.55% and 34.74%, respectively. Those that disagreed with the leader represented 9.74% and 20.53%, respectively.

From the homophily analysis, it is interesting to observe the relation between the variables homophily and interaction by this political leader. Errejón especially addressed favourable comments (48% and 40.74%, respectively) and neutral ones (46% and 37.94%, respectively). Conversely, the attention he paid to disagreeing comments was respectively 6.25% and 22.22%. When homophily was linked with the type of comment, most of those that disagreed had an argument as their basis (78.76% and 58.33%, respectively). One piece of relevant information was the exclusive relation between theoretical comments and a favourable attitude to the political leader's arguments (88.8% and 83.33% of the detected theoretical comments, respectively). In this type of comments (Tables 6 and 7), the “neutral” people opted to use questions during both streamings (33.33% and 34.83%). Nonetheless, rationality was detected only in 30.12% of the comments made (47 of the 156 interventions made as a comment).

**Table 6:** Types of interventions in the chat of the streaming entitled ‘Talking about mental health from Parliament’.

Type of intervention	Count	%
Comment	265	25.43
Question	142	13.63
Personal experience	95	9.12
Laudatory	88	8.45
Critical comment	67	6.43
Gratitude	65	6.24
Greeting	63	6.05
Request	61	5.85
Referring to another user	31	2.98
Unknown	29	2.78
Troll	26	2.50
Comment (theoretical/with an argument)	20	1.92
Specification	15	1.44
Proposal	14	1.34
Humour	14	1.34
Cultural recommendation	7	0.67
Criticism of others	6	0.58
Falling in love	6	0.58
A desire	4	0.38
An offering	4	0.38
Others (values below 4)	20	1.92

**Table 7:** Types of interventions in the chat of the ‘Meritocracy’ streaming.

Type of intervention	Count	%
Question	65	16.93
Critical comment	58	15.10
Comment	51	13.28
Theoretical comment/with an argument	47	12.24
Greeting	39	10.16
Laudatory	30	7.81
Referring to another user	24	6.25
Request	15	3.91
Laughter about another user's comment	10	2.60
Unknown	10	2.60
Gratitude	5	1.30
Cultural recommendation	5	1.30
Proposal	4	1.04
Troll	4	1.04
Others (values below 4)	17	4.43

His reciprocal interaction showed that Errejón answered 4.61% of the comments and 7.03% during each streaming, and he answered the participants using their names. During two chats, 59 messages were repeated (3.74% and 4.31% of those posted during each streaming). In these cases, the user once again copied the same text in the chat. In fact 0.06% of the messages (n = 10) were identically posted on three occasions during chats (n = 6 for the first and n = 4 for the second).

In the respectful discourse analysis, the number of comments made in neutral and respectful language stood out, and respectively represented 95% and 88.80%. Moreover, 49 (10.86% of the total) and 31 (21.09% of the total) accounts, respectively, were noted to make at least five interventions during each live streaming, which means that they could be classified as active users. The references made by users employing vocatives barely represented 2.98% and 6.25%, respectively.

Finally, the references made in comments about foreign nationality and age were minimum. Of those noted during both live streamings, the people in the foreign audience who identified themselves (N = 34) came especially from Argentina (N = 15). The audience's age ranged from 16 to 45 years (N = 15).

## 5 Discussion and conclusions

Our findings allowed us to identify three novel Twitch contributions to digital political communication. Firstly, came his use to reconnect with citizens in accountability terms. Of the power's scrutinising mechanisms, Twitch allows Errejón to set up a new typology: self-monitoring. Here it is the elected politician who proactively exercises accountability with citizens with no third party intervening or anyone making him do this. In the monitory democracy context [26], this social media favours a dynamic to explain political proposals and decisions without the time restriction that exists in Parliament, the spacio-temporal restrictions that apply to other social media (that make communication rushed and fragmented) or the intermediation of news organisations and journalists. Therefore, Twitch enables the monitoring nature to be extended by adding this new format, which contributes to strengthen democracy.

Secondly, Twitch reinforces politicians' authenticity and naturalness. This digital platform favours live long-lasting streaming in which the improvisation motivated by answering the audience's comments predominates. Employing messages related to personal issues, such as musical or gastronomic preferences, which Errejón has done, reveals a use of this digital platform to favour humanising political figures. By showing some of his intimate details, this politician aims to approach citizens and be seen as just another person, someone close. This reveals that Twitch permits mediated authenticity to be activated as a key value in the political actor's public construction [30]. This favours connections being established with citizens. It also contributes to acknowledge other people because Errejón mentions his followers by name whose questions he answers to seek engagement and a link with citizens.

Thirdly, our results suggest using Twitch to promote new political and communication practices linked with connective democracy. The fact that several deliberative characteristics are present on Errejón's channel suggests that he attempts to promote a two-way communication model by encouraging dialogue and conversation with the audience via his channel. This is an attempt to outdo the preeminent communication model on other digital platforms like Twitter or Instagram [2,5] based on a passive public who can be influenced by electoral persuasion, and one centred on merely diffusing information and contents via one-way communication. By being present on Twitch, Errejón achieves a new way to relate with the public in an attempt to connect the political-side with the citizen-side by means of interactive communication. Far from renouncing dialogue, he attempts to stimulate dialogue by seeking cross-cutting deliberation in which people with different ideological points of view can participate. The percentages of favourable messages sent to Errejón, and the fact that most involve respectful discourse, reveal that homophily is moderately present in an ample space for both disagreement and debate. And so it is that the Habermasian standard of discourse diversity is met. This opens up the way to reduce the social divisions caused by polarisation and political discontent by following connective democracy postulates [23,39]. By means of such deliberative uses, reconnecting citizens is feasible [40] for generating benefits for democratic health by taking an engagement-centred approach.

Our findings suggest that this type of political use on Twitch has the potential to generate new connections with citizens. Nonetheless, several requirements are necessary if it is to be effective. Firstly, this politician makes a commitment about the frequencies with which he will appear. Errejón's first streaming managed to gain the audience's loyalty, but that the audience lost interest in him when he did not meet his frequency commitment. Secondly, debate must be about a main subject, and should not cover particular interests, so that a topic can be dealt with in depth. Finally, the third requirement is that not any exchange of views can be accepted during this interaction, but extra rationality in arguments must be prioritised, as must verifying the exactness of data [70]. Errejón must base his speeches on the principle of parrhesia [71].

However, Errejón's political use of Twitch has its limitations. The main one is that rationality was barely demanded. The majority of comments were generalist comments and questions, and included personal experiences with neutral content that evidenced the audience's depoliticisation and its need to be heard. Therefore, this platform would help the sectors that do not tend to use political information, especially young people, to show an interest in it [[72], [73], [74]].

The present research work reveals that political entrepreneurs can innovatively use digital platforms, such as Twitch in our case, which activate communication strategies with the potential to establish connections with citizens, and to do so in a deliberative and monitory way. In a crisis context, and one that is currently being redefined (e.g. today's context), this is a valuable contribution to maintain and strengthen democracy. The present work is not without some limitations. It is the first approach to explore Twitch being employed as a digital political communication tool. The fact that only a few relevant politicians use this platform for political goals makes taking a comparative approach difficult. Despite its important contributions, our work would have been enriched with a comparative analysis of several cases. Furthermore, our approach centres on studying the broadcaster. This leaves some issues pending for future research that are related to not only the motivations and attitudes of the citizens who participate on Errejóns’ Twitch channel, but also to possible consequences on their political conducts and visions after this experiment. This would supplement our knowledge about the political incidence of this digital platform and its democratic potential.

## Fundings

The work of Andreu Casero-Ripollés is supported by grant number PID2020-119492 GB-I00 funded by MCIN/AEI/10.13,039/501,100,011,033/ and “FEDER A way of making Europe” and by grant number UJI-B2020-14 funded by the Universitat Jaume I de Castelló within its Research Promotion Plan.

The English translation of this article has been funded by the Conselleria d’Innovació, Universitat, Ciència i Societat Digital of the Generalitat Valenciana aimed at the celebration of the ‘Dissent and communication: voices and discourses in the era of alternative facts' conference (Reference: AORG/2021/025).
